# Reformulated Object Relations Theory: A Bridge Between Clinical Psychoanalysis, Psychotherapy Integration, and the Understanding and Treatment of Suicidal Depression

**DOI:** 10.3389/fpsyg.2021.721746

**Published:** 2021-09-22

**Authors:** Golan Shahar

**Affiliations:** Department of Psychology, Ben-Gurion University of the Negev, Be'er Sheva, Israel

**Keywords:** clinical-psychoanalysis, object-relations-theory, positions, suicidal-depression, psychotherapy integration

## Abstract

In contrast to the fruitful relationship between psychoanalysis/psychoanalysts and the humanities, institutionalized psychoanalysis has been largely resistant to the integration of psychoanalysis with other *empirical* branches of knowledge (infant observation, psychotherapy research, psychological and neurobiological sciences), as well as clinical ones [primarily cognitive-behavioral therapy (CBT)]. Drawing from two decades of theoretical and empirical work on psychopathology, psychotherapy, and psychoanalysis, the author aims to show how a reformulation of object relations theory (RORT) using (neuro-)psychological science may enhance a clinical-psychoanalytic understanding and treatment of suicidal depression, which constitutes one of the most formidable health challenges of our time. Specifically, he rewrote the notion of Melanie Klein *positions*—primarily the depressive position—using extant knowledge of structure of emotions, the centrality of mental representations of the future (“prospection”) and the toxic nature of criticism-based emotions. This reformulation enables a dialog between clinical psychoanalysis and other therapeutic schools of thought and sheds light on the understanding and treatment of suicidal depression.

Psychoanalysis has always been comfortable conversing with the humanities (Freud, 1930; Kohut, 1980; Jurist and Orfanos, 2016), as well as with the more hermeneutic branches of social sciences (e.g., Freud, 1921; Paul, 1989; Powell, 2010). By the same token, psychoanalysis has had acrimonious relationships with empirical, quantitative science, whether medical or (neuro-)psychological (Blass and Carmeli, 2007; Bornstein, 2007). Like many others, the author has repeatedly lamented this conflictual relationship between psychoanalysis and empirical science (e.g., Shahar, 2010, 2015a). In this article, the author seeks to examine one of the most unfortunate consequences of the psychoanalysis-empirical science rift: the difficulty in pursuing a dialog with other schools of psychotherapy, primarily the cognitive-behavioral, manualized-interpersonal, emotion-focused, and family-systems perspectives. To be sure, the past two decades have seen several examples of successful bridging and integration, as is shown by the development of evidence-based psychoanalytic treatments such as transference-focused therapy for borderline personality disorders (TFT; Yeomans et al., 2013), the development of psychodynamic-integrative treatments (e.g., Beutel et al., 2019), the incorporation of psychoanalytic ideas in other treatment approaches (for instance, in Schema Therapy; Refaeli et al., 2010), and collaborative research studies involving proponents of different theoretical approaches (e.g., Levy et al., 2006). These important advancements, however, are as scarce as they are scattered. This article is offered in an effort to extend these advancements and to apply them to a gnawing problem faced by clinicians from various disciplines and perspectives, namely, suicidal depression.

## The Psychoanalysis-Science Rift: A Precursor of the Seclusion of Psychoanalysis From Other Therapeutic Perspectives

A large part of the seclusion of psychoanalysis, particularly clinical psychoanalysis, from other therapeutic perspectives is the traditional animosity of psychoanalysis toward the very notion of empirical research (e.g., Kernberg, 2006). One of the ramifications of this animosity is the reluctance of the most dominant strands within psychoanalysis to embrace social-cognitive nomenclature of inner (psychological) processes.

The social-cognitive lexicon is primarily cognitive. Terms such as schemas, scripts, biases, attitudes, information-processing, problem-solving, decision-making and the like, are the offspring of the cognitive revolution in academic psychology (see Miller, 1951, 2003), a revolution that essentially ended the anti-mentalistic thrust of radical behaviorism (Skinner, 1957, 1971). Theoretical and empirical works of Albert Bandura, Walter Mischel, Daniel Cervone, and their followers have “interpersonalized” the aforementioned cognitive concepts (Mischel, 1973; Bandura, 1986; Cervone, 1991), showing how cognition actually serves as the glue of social transactions. These works of Bandura, Mischel, Cervone, and their colleagues echoed the ascendance of attachment theory into prominence within academic psychology. Commencing with the works of Bowlby (1969) of the Independent Group of the British Psychoanalytic Society (to which Winnicott also belonged) and facilitated by the subsequent works of Ainsworth and Bell (1970) and Mary Main and colleagues (Main et al., 1985), attachment theory has evolved around the understanding that actual (as opposed to fantasized) parent-child relationships create *internal working models* (IWMs) of relationships. These IWMs organize actual relational patterns throughout the life span (Mikulincer and Shaver, 2016; Fonagy and Luyten, 2018) and account for differential interpersonal patterns surfacing in close relationships (Bartholomew and Horowitz, 1991).

The contribution of this social-cognitive lexicon to psychological science is 3-fold. First, the concepts included in this lexicon are very clear, even to scholars coming from outside of psychology. Second, these concepts are empirically testable. Third, the endeavor of testing these concepts has bolstered the development of research procedures that, in turn, facilitated empirical research both within and outside of psychology (e.g., priming, Kahneman, 2011). In light of this, a select few within academic psychology who are also psychoanalytically oriented have compellingly demonstrated that the most profound psychoanalytic concepts may be described in social-cognitive terms (e.g., Blatt, 1990, 1995a; Westen, 1991, 1998; Fonagy and Allison, 2012). Unfortunately, despite the impressive accomplishment of these giant academics/psychoanalysts, from a sociology-of-science standpoint, their efforts to bridge the psychoanalysis-science rift have yielded limited success (Bornstein, 2001; Shahar, 2010).

Interestingly, at the same time, that psychoanalysis braced itself against “scientization” (Hoffman, 2009), other clinical schools of thoughts readily embraced the social-cognitive nomenclature, thereby forming a vibrant alliance with academic psychology. The obvious exemplar is cognitive-behavior therapy (CBT), which—after essentially translating social-cognitive science into straightforward clinical interventions—has succeeded in branding itself as the preeminent evidence-based approach to psychotherapy. This claim, alas, is not always in the best interest of their patients (Swedish National Audit Office, 2015; Dalal, 2019). In addition to CBT, family-systems, experiential-gestalt, and humanistic-existential psychotherapeutic perspectives also embraced the social-cognitive nomenclature. This enabled these perspectives to gain respect within academic psychology (see, for instance, Seligman et al., 2013; Fosco et al., 2016), and thus be considered seriously in published guidelines for psychological treatments for a wide range of mental disorders (e.g., Guideline Development Panel for the Treatment of Depressive Disorders, 2019)^1^. This pattern is particularly noteworthy with respect to the humanistic-existential perspective, which shares with psychoanalysis a fascination with the humanities and a philosophical/romantic writing style (see Strenger, 1989), but which has always played a major role within academic psychology and evidence-based psychotherapy (Shahar and Schiller, 2016a).

## Psychotherapy Integration: An Ideal Arena for the Dialog Between Psychoanalysis and Other Approaches

Despite the seclusion of psychoanalysis, an arena has been forming since the late 1970's, which appears to serve as the ideal locus for a potential dialog between psychoanalysis and other psychotherapeutic schools of thoughts. This arena is the psychotherapy integration movement (Ziv-Beiman and Shahar, 2015), spearheaded by the Society for Exploration of Psychotherapy Integration (SEPI; https://www.sepiweb.org/). Founded by clinicians, researchers, and theorists from diverse persuasions, SEPI is an international and interdisciplinary organization aimed at bridging across diverse therapeutic orientations. Some of many illustrious founders of SEPI, such as George Stricker and Paul Wachtel, and current leaders are prominent psychodynamically oriented academics/clinicians. It is, therefore, of no surprise that SEPI and its flagship *Journal of Psychotherapy Integration* (JPI) are psychoanalytically friendly. But what is more important is that, by design, SEPI and JPI push the psychoanalytically oriented members to get to know, appreciate, and learn from other schools of thought in psychotherapy.

Unfortunately, however, SEPI and its resources are largely ignored by the most dominant strands of the psychoanalytic movement. To relate an anecdote as evidence: in Israel, where the author lives and practices, there is a huge interest in SEPI and its integrative mission. But this interest, manifested by clinicians from numerous persuasions (including psychodynamic), largely escapes the Israeli psychoanalytic establishment. The author has repeatedly heard prominent figures within the Israeli psychoanalytic establishment ridicule the integrative mission and dismiss it as “just another version of CBT.”

## RORT: A Conceptual Framework for the Psychoanalysis-Social Cognition Dialog

If we were to imagine that institutional psychoanalysis would suddenly change its ways and work to engage with the academic, evidence-based social-cognitive nomenclature, a question would quickly arise: Which strand of psychoanalysis?

Psychoanalysis is characterized by having numerous schools and strands (e.g., Ghent, 1989), and it is in the habit of each school to deem the others “non-psychoanalytic” (Blass, 2010). This factor renders an answer to the above question difficult to arrive at. For the sake of the present discussion, however, let us assume that the largest strands of psychoanalysis are: (1) the classical/Freudian, which is drive-focused, (2) psychoanalytic ego psychology, (3) object relations theory and attachment theory, (4) psychoanalytic self-psychology, and (5) interpersonal/relational psychoanalysis. Which of these should be “socially cognitivized?” Interestingly, all five strands enjoy supportive research evidence, the review of which lies outside the scope of this article. Selection, therefore, should be made on a conceptual basis. My contention would be that most prominent strand of psychoanalysis, and the one that is most promising in terms of conversing with academic psychology, is object relations theory (e.g., Greenberg and Mitchell, 1983), particularly when it is linked with attachment theory (Levy et al., 1998; Levy and Blatt, 1999; Fonagy et al., 2018).

The rationale for my selection is that, from a philosophical or metapsychological perspective, object relations theory constitutes the most comprehensive theoretical statement of psychoanalysis, one that subsumes each of the other four strands, and that it lends itself most easily to clinical practice that is conversant with non-psychoanalytic schools. Specifically, ORT recognizes biological drives as central to human psychology and action, while drawing primarily from Melanie Klein, highlighting that the drives are invariably directed toward human figures. Furthermore, ORT acknowledges the principal role of the ego as a self-sector responsible for regulating thought, affect, and behavior, although the ego, as a self-sector, is always *in-relationships*. ORT can easily incorporate psychoanalytic self-psychology by acknowledging the ability of the latter to elucidate the unfolding of narcissistic phenomena (Blass and Blatt, 1992). Furthermore, as extensively argued by Mitchell (1995a) and others, the interpersonal/relational psychoanalytic strands, as much as they are adept in describing interpersonal behavior, stand upon the description of ORT of mental representations of self and others. Finally, ORT is very strongly represented within academic psychology, not only through seminal works by Westen (1991, 1998) and Blatt (e.g., Blatt et al., 1997) but also through the voluminous empirical and theoretical literature on attachment theory (Mikulincer and Shaver, 2016; Fonagy and Luyten, 2018), with its emphasis on internal working models (IWMs), a term largely equivalent to “object relations” (for similarities and differences between ORT and attachment styles, see in particular Levy and Blatt, 1999; Blatt and Levy, 2003; Shahar et al., 2004a; Fonagy and Luyten, 2018; Fonagy et al., 2018).

Building on the intellectual accomplishments yielded by ORT, my theoretical and clinical work over the last two decades has focused on bridging this theory with more recent developments within academic psychology (e.g., Shahar, 2001, 2004, 2006a, 2010, 2011, 2012, 2015a,b, 2016, 2018; Shahar et al., 2004a; Shahar and Davidson, 2009), particularly cognitive psychology, existential philosophy and psychology, neuroscience, and research on self and consciousness. The major thrust of this work is reformulating the notion of Klein (1928, 1935, 1940, 1945) of the positions.

Readers of articles in this special issue are hardly in need of an exposition of the positions, but I nonetheless offer here a brief one. According to Klein, a position is *a system* comprised of object relations, defense mechanisms, and specific anxieties. She states: “With the changes in the relation to the object, new anxiety- contents make their appearance and a change takes place in the mechanisms of defense” (Klein, 1935, p. 146). Thus, the three components do not just co-exist, they bolster and augment each other.

With respect to anxiety, Klein (1928) and her followers distinguish between a *paranoid-schizoid* anxiety centered around a fear of overwhelming aggression and a *depressive* anxiety evolving about the fear of harming the good. Per defense mechanisms, Kleinians distinguish between primitive defenses aimed at keeping the good at bay from the destructive influence of the bad (e.g., splitting, projective identification, and idealization) by polarizing good and bad, and the more neurotic defense mechanisms which aim at pushing inner flaws out of awareness. With respect to object relations, Kleinians underscore the difference between *part* object relations, whereby self and others are represented as either good or bad, and *whole* object relations, whereby both self and others are likely to contain multiple, good *and* bad, characteristics. In this theory, the paranoid-schizoid position consists of paranoid anxieties, primitive defense mechanisms, and part object relations. This position accounts for severe psychopathological syndromes, such as psychosis and borderline personality disorder. Conversely, the depressive position consists of depressive, guilt-ridden anxiety, neurotic (moderate and nuanced) defense mechanisms, and whole (ambivalent) object relations.

As the author stated previously (Shahar, 2018), Klein's notion of the positions are imbued with profound insights into the human condition, which make them particularly useful for the following reasons:

(1) The positions chart psychological development as characterized by *increasing cognitive and affective complexity* (i.e., the more primitive paranoid-schizoid position is succeeded by the more nuanced depressive position).
(2) At the same time, unlike stage theories of development (e.g., that of Freud and Piaget), once a new position is formed, it does not nullify the previous one. They coexist, with a single position occupying the center of the psyche while the other operates in the background. This coexistence of the positions enables very rapid oscillations between diverse levels of personality organization, so rapid that individuals may appear, within hours, at first highly unstable and hostile and then composed and collected, or *vice versa*. Stage-like notions of personality progression and regression do not so well-account for the existence of such rapid shifts in personality organization.
(3) The notion of positions epitomizes the fact that, in the human personality, various aspects and processes work in tandem: A unidimensional nature of paranoid anxiety necessitates dramatic defensive measures aimed at keeping the good away from the bad (e.g., splitting), and such dramatic defensive measures can only be executed by a self that is full of conviction as to who is good and who is bad. In contrast, the depressive, guilt-ridden anxiety inherently recognizes the coexistence of good and bad (Eros and Thanatos) within the self, requiring more circumscribe defensive measures that cloud specific self-aspects (e.g., repression, displacement, and reaction formation), as opposed to severing large self-segments. Put differently, a multifaceted structure of self and other representations disallows a unidimensional, paranoid anxiety.
Why, then, the need to reformulate the positions? For two reasons. First, the Kleinian jargon is problematic. “Good and bad breast,” “projective identification,” these terms are not just technical. They virtually alienate scholars coming from outside psychoanalysis and, as I can personally attest, even several scholars coming from within. Secondly, and not necessarily unique to the notion of the positions, their description is completely removed from knowledge accumulated through scientific methodologies for decades. I am referring specifically to knowledge about emotions, awareness, and the self.

### Nuanced Emotions

In most psychoanalytic theories, including that of Klein, anxiety epitomizes negative affect. Hence, in these theories, anxiety is the emotional state imbuing most, if not all, dynamic consequences. To paraphrase Aristotle, anxiety is the prime mover of the psyche. This, however, is not the way emotions seem to work. The larger category of negative affect is much more nuanced, comprised of numerous emotions that are aversive. The obvious ones are sadness, anger, and guilt, but one can easily add to these three emotions, such as shame, disgust, disappointment, contempt, hostility, and irritability (Watson et al., 1988). Moreover, positive emotions appear to be largely independent of negative emotions, which means that people may experience both negative and positive emotions *concurrently* (e.g., Tuccitto et al., 2010). For instance, while writing these words, the author was both curious and enthusiastic (because he is interested in the topic and geared toward deciphering it) and nervous and fearful (that he would not do a good job explicating his ideas). After completing this work, he was likely to feel a measure of pride but also mental exhaustion and some emptiness.

### Awareness of a Continuum

Kleinian psychoanalysis, arising from classical formulation of Freud, rests on the idea of a thick “repressive line” that squarely separates conscious from unconscious material (Billig, 1999; Fink, 2009). Along what Ricœur (1970) labels “the hermeneutics of suspicion,” unconscious material is more real, and is also much more difficult to come by compared with conscious material (see Eagle, 2011).

Interestingly, this “hermeneutics of suspicion” is starkly inconsistent with research evidence attesting to the ease with which threatening mental material pushed outside of consciousness may then be summoned back based on experimental manipulation (e.g., Tzelgov, 1997; Erdelyi, 2006; Shahar, 2006b; Sedikides and Green, 2009). Consider, for instance, the *mnegic neglect effect* (e.g., Sedikides and Green, 2004), whereby—as shown in the psychological laboratory—people selectively forget feedback that threatens their self-concept. However, a simple experimental procedure (“priming”) aimed at increasing accessibility to self-improvement motives completely erases the mnegic neglect effect (Green et al., 2009).

Such experimental evidence tallies with gradual transformations in psychoanalytic theory, which eschew this notion of a clear divide between conscious and unconscious materials. Beginning with Ferenczi, continuing with Balint, Winnicott, Guntrip, and Kohut, and being represented contemporaneously by Stolorow et al. (1987), Strenger (1989), and Eagle (2011), these theorists and others adopt a more continuous, experience-near, phenomenological perspective whereby material repeatedly, possibly very rapidly, oscillates between conscious and unconscious levels, often in a way that is dependent upon central life goals of patients. For instance, in psychotherapeutic work of the author with first-time parents, he is impressed by the fact that this role transition summons memories and mental material—often painful—concerning the early relationships of patients with their own parents. Such material has always been *known* to these first-time parents, although quite often, it has not entered into the center of awareness, and was thus left unprocessed. This material becomes highly accessible when patients turn into parents, arguably because it is now needed to (mis)guide their central developmental task^2^.

Why is it important to replace the thick repressive line perspective on awareness with one that is continuous/phenomenological/experience-near? Because the latter opens pathways to understanding the positions. In stress theory and research, there is a traditional distinction between defense mechanisms and coping strategies (Haan, 1977; Cramer, 2006; Vaillant, 2011), which is based on the notion that defense mechanisms are unconscious whereas coping strategies are conscious. However, adopting a continuous/phenomenological/experience-near perspective essentially nullifies this distinction, in turn encouraging the consideration of both defense mechanisms and coping strategies as partly conscious ways to regulate affect. In fact, the author posits that the term *affect regulatory strategies* should be used instead of defense mechanisms in describing specific positions. The author illustrates it below when outlining a reformulated depressive position.

### A “Projected” Self

The vast majority of psychoanalytic theorizations of self and “objects” focus on the impact of the past—particularly early relationships with caregivers—on object relations and on the reactivation of these object relations in the present and in the interpersonal arena. In contrast, the future tense is quite neglected in psychoanalytic theory. This is as interesting as it is unfortunate, given that the bulk of empirical research amassed over the last four decades now clearly points to the centrality of the future in the psyche (Seligman et al., 2013). Specifically:

1. Mental representations of future goals and “projects” appear to have a profound impact upon our behavior (Austin and Vancouver, 1996);
2. A form of cognition called *prospective memory* has been discovered, pertaining to memory for actions planned to be performed in the future (McDaniel and Einstein, 2007). Prospective memory is considered a cognitive faculty, which is highly important for everyday life;
3. Several brain areas (e.g., Brodmann Area 10 at the prefrontal cortex, PFC) and processes are considered to be in charge of future planning;
4. Theory and research comparing humans with other species suggest that the principal function of the PFC, an area largely unique to humans, is to plan for the future (Amati and Shallice, 2007);
5. Representations of the self in the future (future self) predict behavior in general, and behavior aimed at influencing the future (e.g., saving for retirement) in particular.

Here, as in the case of awareness, this slew of empirical research is consistent with small, albeit insistent, streams of psychoanalytic theorizing that does touch upon the role of the future in the psyche (Summers, 2003). Consider, for instance, worlds of Sullivan (1953):

“*If he is interested in psychiatry, he is almost certain to come to consider the role of foresight in determining the adequacy and appropriateness of the energy transformations, his overt and covert activity, with respect to the actual demands of the situations in which he finds himself involved with significant others* (Sullivan, 1953, p. 369; italics in the original).

And, later, on the same page:

*I am saying that, circumstances not interfering, man the person lives with his past, the present, and the neighboring future all clearly relevant in explaining his thought and action; and the near future is influential to a degree nowhere else remotely approached among species of living*. (Sullivan, 1953, p. 369; italics in the original).

In a contribution entitled “The Future as Intrinsic to the Psyche and Psychoanalytic Theory,” Summers (2003) reviews the scant treatment of the future in psychoanalysis. For instance, he mentions emphasis of Loewald on expectations of the superego, language of action of Schafer, in which the self-narrative includes future goals, notion of Bolas of destiny (which the author believes is quite similar to the humanistic notion of self-actualization), and others. In applying this review to the clinical situation, Summers (2003) states:

“It follows that the analyst who looks at time in only a linear fashion, in which the past affects the present and future, adopts a simplistic and limited view of temporality that does not fit the lived experience of time. Because the present moment is embedded in and only gains meaning in the projected future, or pluperfect tense, understanding the patient's present requires that the analyst grasp the patient's experience of the future and how the present moment fits into it. The emptiness, passivity, and complacency we see in so many patients reflect their loss of the future, an inability to live in the pluperfect tense, and this empty future issues in the bleakness of their present lives. Because the present and past gain their meaning via their relationship to the projected future, or the pluperfect tense, when the future looks dim, the present becomes empty and the past constricting. When the future looks bright, the present shines and the past is viewed as potentially useful, as a way to transform the present. To be sure, we can all look at the past and find reasons why the future looks so bleak and the present empty, but it is equally true that the void in the future leads to an empty present and a sense of imprisonment in the past. (Summers, 2003, pp. 139–140)”.

This relatively recent theoretical thrust within psychoanalysis, consistent with empirical research, draws psychoanalysis closer to philosophical and psychological existentialism (May, 1958; Cooper, 1999; Shahar, 2010), with its emphasis on authenticity and goal-directed action.

This brings us to integrating the above issues for the sake of reformulating the position of Klein. Here is the proposed definition of the author:

A position is an amalgamation of affect, its regulation, and schemas and scripts of self-in-relationships, all of which augment one another and form a distinct and coherent experience of the world. Positions are formed throughout childhood and adolescence, and are maintained *via* interpersonal action. They are projected into the future, representing the hope and fears of an individual, and, as such, are guiding cognition, motivation, emotion, and behavior. Although all positions strive to be confirmed in the interpersonal arena, those positions, which occupy a large space in the psyche, and which are trauma based, are likely to create a maladaptive social environment that culminates in psychopathology.

Breaking down the key segments of this definition, we may note that:

1. Positions are causal systems that include reciprocal influences of affect, affect regulation, and schemas and scripts. Put differently, positions epitomize the perspective whereby affect, motivation, and cognition are all co-causative (Beck, 1996).
2. The evolutionary advantage of positions is the clarity they afford. The humans need a clear worldview so that they can know how to act (Amati and Shallice, 2007; Shahar, 2015b). The subjective experience of the position is vivid and could be given the following words: “This is what the world looks like, and I should act accordingly.”
3. Rather than being merely mentalistic, positions are translated *into action*, whereby people try to shape reality in accordance with their worldviews. Thus, positions that include persecutory features are likely to translate into enemy-making actions, whereas positions, which include optimistic features, are likely to lead to positive outcomes. Concepts such as self-fulfilling prophecy, a vicious or virtuous cycle, positive feedback loop, and dynamic equilibrium are all consistent with this characteristic of the positions (see, in particular, Wachtel, 1994).
4. As indicated in items 2 and 3 on this list, positions are projected into the future. The future, therefore, is a good place to start from in an attempt to understand and modify positions (Shahar, 2011, 2012).
5. There are likely to be many more positions in the psyche than the two identified by Melanie Klein (and of these two, at least one, the depressive, needs an extensive reformulation, offered below). The author can think of paranoid-schizoid, depressive, obsessive, somatic, dissociative, playful/humoristic, and stress-resisting positions, among others. From an evolutionary point of view, we can say “the more the merrier,” because many positions increase the repertoire of adaptive behavior of an individual, particularly under threat. However,
6. There is a possibility that a single position may “eclipse the others,” in that it occupies a disproportionally large segment of the psyche. When this happens, a concerted—albeit only partially conscious—effort is made by the individual to confirm this position in the interpersonal arena, and this effort is likely to be successful. When the interpersonal arena is shaped according to the position, psychopathology ensues. The distinct nature of the psychopathology (e.g., anxiety, depression, eating disorders, somatization, and psychosis) “speaks,” by means of symptoms, the inner drama of the positions.

## The Reformulated Depressive Position and its Utility in Treating Suicidal Depression

My career, both academic and clinical, is largely devoted to neutralizing clinical depression, an affliction which the World Health Organization (World Health Organization, 2012) considers a major pandemic of our time, even more so during and after the current COVID-19 pandemic (Ettman et al., 2020). Although depression has been studied extensively, and despite the development of numerous empirically supported therapeutic protocols developed to treat the disorder (including psychodynamic psychotherapy), depression still spreads around the modern world (Rottenberg, 2014; Dowds, 2018). Its heterogeneity (i.e., that individuals with different symptomatic profiles might be similarly diagnosed as depressed (Coyne, 1986; Monroe and Anderson, 2015), relapsing-recurring course (Kessler et al., 2005), medical, educational, and economic complications (Blumental and Lett, 2003; Lépine and Briley, 2011), and the fact that it is potentially lethal [i.e., suicide (Joiner, 2007)] renders this clinical condition an ever more formidable challenge to researchers, practitioners, and policy makers.

It would, therefore, be natural for me to consult writings of Klein, particularly those concerning the depressive positions, in order to understand this ubiquitous disorder. However, as the author has previously argued (Shahar, 2018), a close reading of work of Klein on depression reveals that, for her, clinical depression is an obscure construct. Klein was at her best describing psychotic-like and other “primitive” psychopathological conditions, while her description of depression sometimes gives the impression that the condition is, actually, an indication of *health*. She merely hints at psychological processes that render individuals vulnerable to depression (e.g., failure in repairing a good object harmed by actions of the self) but rarely, if ever, explains this vulnerability, and how it translates into symptoms (see also Ogden, 1992, for whom the “depressive” position is actually a “historical” position, representing an achievement of a normal personality organization). Indeed, when Klein writes about clinical depression, the writing quickly “regresses” into paranoia and/or mania (e.g., Klein, 1935, p. 158).

**Figure 1:**
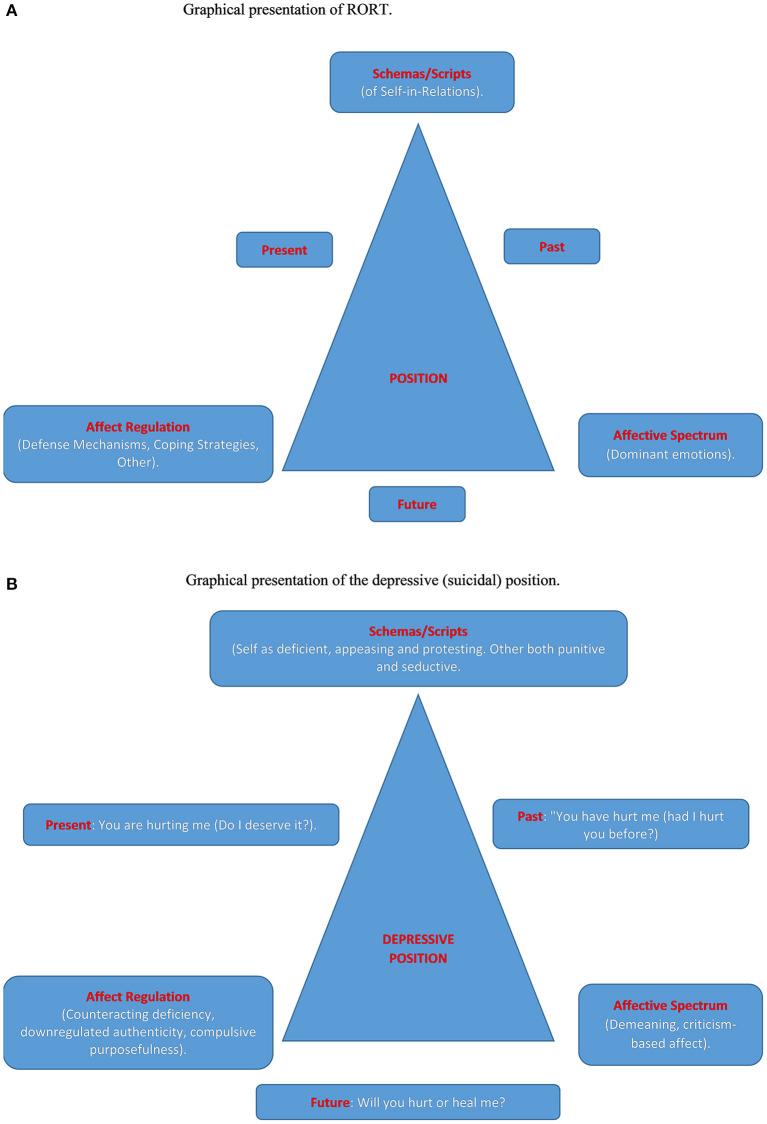
**(A)** Graphical presentation of RORT. **(B)** Graphical presentation of the depressive (suicidal) position.

On the other hand, when my reformulation of the Kleinian positions is applied to the extant knowledge of depression, new avenues are opened in the understanding of the disorder, and, particularly, the path leading from depression to suicidality. In Figure 1A, the author presents a graphical summary of RORT and, in Figure 1B, a graphical summary of the reformulated depressive/suicidal position. This summary goes beyond his previous formulation of this position (Shahar and Schiller, 2016b; Shahar, 2018).

To understand how the author crafted Figure 1B, one should get acquainted with some key findings concerning the nature of unipolar depression. In particular, and consistent with cyclical psychoanalysis of Wachtel, a consensus is being formed among clinicians and researchers alike that depression is profoundly interpersonal (Pettit and Joiner, 2006). That is, people with depression actively, if inadvertently, create the very interpersonal, social conditions that are implicated in the onset, relapse/recurrence, and maintenance of the disorder. Life stress is a prototypical example. Stressful life events, such as a breakup or divorce, job loss, loss of a loved one, and other “exit events” (Paykel, 2003), have been demonstrated for decades to predict the depressive onset and recurrence (Brown and Harris, 1978). However, mounting evidence suggests that rather than constituting a *force majeure*, stress might actually be propelled by the depressed persons themselves (Hammen, 1991). Other social and interpersonal factors that are implicated in depression and might be generated by the depressed person are lack of social support (Dew and Bromet, 1991) and lack of positive life events (Bylsma et al., 2011).

But is it depression *per se* that is actively maintaining itself? The works of (in alphabetical order) Blatt, Dunkley, Gilbert, Shahar, Zuroff, and others suggest that behind this active maintenance of depression lies a formidable force called *self-criticism* (Blatt, 1995b). Defined as tendency of individuals to set unrealistically high standards for performance and to “bash oneself” once these standards are not met, self-criticism has been shown to generate stress, “degenerate” social support and positive events, and to erode therapeutic relationships. All of these effects are demonstrated even when depressive symptoms are statistically controlled. In fact, in many of these studies, when the propagating research findings of depression and self-criticism were compared, it was the former, rather than the latter, that produced depressogenic interpersonal conditions (see Shahar, 2015b, 2016, for review). Moreover, depressive symptoms and related psychopathology might also prospectively predict an increase in self-criticism over time (e.g., Shahar et al., 2004b; Schiller et al., 2016; Shahar and Henrich, 2019). This suggests that depression and self-criticism are co-causative, constituting a “self-critical cascade” (Shahar, 2015a, 2016). The author suggests that the reciprocal relationships between self-criticism and depression be viewed as a segment of *a larger system of mutually causative elements characterized by affect and cognition*, that is, the depressive position (reformulated).

Research has shown that self-criticism activates a host of other emotions besides sadness, which is prototypical for depression. Shame, anger, and contempt might constitute such affective states (Whelton and Greenberg, 2005). Moreover, self-criticism and painful affect might be reciprocally related through maladaptive defense mechanisms such as acting out, undoing, projection, devaluation, denial, isolation, splitting, and turning against oneself and others (Besser, 2004), as well as more conscious, maladaptive coping strategies, such as venting distress to others without attempting to solve the putative problem (Dunkley et al., 2003), and highly maladaptive motivational regulative endeavors, namely, attempting to suppress authentic interest in activities (Shahar et al., 2003). Some of the aforementioned defense mechanisms, notably projection, turning against others, and splitting, actually shed light on a very close link—also likely to be reciprocal—between self-criticism and representations of other people. Specifically, self-criticism is shown to be strongly associated with the perception of others as harsh, punitive, and judgmental (Mongrain, 1998).

Synthesizing this voluminous line of empirical findings, the author proposes the following:

### Affect

The most dominant affect in the reformulated depressive position is neither anger nor sadness but, rather, a demeaning, criticism-based affect. It consists of emotions, such as anger, shame, guilt, content, disgust, disappointment, hatred, and envy. The overall affective tone is that of putting down (either self or other) and focusing on deficiencies.

However, the author proposes that, within this toxic emotional solution, there exists a single emotion that is considered positive, namely, hope. The rationale for this postulate is as follows. Bearing in mind the very rare (and obscure) exception of psychotic depression, unipolar depression is not accompanied by a severe impairment in reality testing. Depressed individuals know at a very basic level what they and the world are made of. This means that people with depression are aware of their strengths and virtues, even though they focus on their deficiencies. In fact, it is this focus on deficiency alongside an awareness of the strength of one that leads to hope: If I succeed in doing/accomplishing this or that, I will be exempt from feeling so deficient.

### Affect Regulation

What are the defenses, coping strategies, and other regulatory maneuvers that are utilized in the depressive position? The author posits that the long list of regulatory maneuvers that have been identified by previous research should be grouped into three regulatory clusters: *counteracting deficiency, downregulating authenticity*, and *maintaining hope through “prospection.”*

*Counteracting deficiency* is straightforward. Experiencing oneself as deficient is incredibly painful, particularly in Western cultures that elevate competence and appearance (hence, the depression-modernity link). Accordingly, a host of maneuvers (defenses, coping strategies) are employed in the service of counteracting deficiency. Some maneuvers are aimed at freeing the self from deficiency, perhaps through turning attention away from it (displacement) or tying it to others (projection, turning against others; Besser, 2004). Another way of counteracting deficiency is inciting guilt in others. This may be done *via* turning against the self so as to seek reassurance (Joiner, 1994; Joiner et al., 1999), manifested *via* coping strategies such as venting distress (Dunkley et al., 2003). Another way to counteract deficiency is to rule it out, convincing self and others that one is wonderful and/or seamless. Klein (1940) has referred to this maneuver as the manic defense (see also Winnicott, 1958; Ogden, 1992; Barett, 2006), which consists of denial of difficulties and threats, disavowal of mellow sentiments, such as loneliness, and inflating self-importance while putting others down.

Another set of regulatory maneuvers is *downregulating authenticity*. Why would that be important? Because, as was so compelling argued by Winnicott (1965) in his treatise of the true self, as well as by Rogers (1963) in describing organismic valuing, authenticity is inherently *spontaneous*, rendering it *unpredictable*. If my inner world is unpredictable, I cannot prevent a situation where I will be caught deficient in some respect. I therefore should always be prepared, which means that I should beware of *going-on-being* (Winnicott, 1960): When the cannons are heard, the muses are silent.

The author and his colleagues conducted a study on self-criticism and motivation (Shahar et al., 2003), demonstrating just this. We assessed 900 American adolescents with regard to their personality, depression, and sources of motivation in both the academic and social domains. We found an unusually strong negative association between self-criticism and “autonomous motivation,” a term developed within self-determination theory (Deci and Ryan, 1985, 2000; Ryan and Deci, 2000), which pertains to doing things because one wants to do them (true self, organismic valuing): The higher the self-criticism of the adolescents, the lower their “autonomous motivation.” This association was held even after controlling for depressive symptoms, and it completely accounted for the adverse effect of self-criticism on positive life events (Shahar et al., 2003, Shahar and Priel, 2003; Shulman et al., 2009).

How is authenticity being downregulated? Primarily by a selective use of isolation of affect (Freud, 1926; Schafer, 1954), directed toward emotions such as joy and enthusiasm. In the cognitive-behavioral world, such a maneuver will be labeled “experimental avoidance,” and research has attested to the link between this construct and self-criticism (Moroz and Dunkley, 2019).

Finally, there is the issue of maintaining hope. How can I keep hope from being annihilated altogether by all these horrible experiences? I can do this by firmly believing that the positive prospect I desire can arrive as a consequence of exact planning and hard work. If I only plan my actions meticulously, I can accomplish enough for others to deem me as non-deficient, hence worthy of love. Thus, defenses, such as intellectualization and rationalization (e.g., Kyle, 2014), are added to the above isolation of affect (joy, spontaneity, and enthusiasm), and they will be used in the service of what I referred to as “compulsive purposefulness” (Shahar et al., 2020), a constant pursuit of worthy goals. Horney (1950) would call it the “tyranny of the shoulds.”

### Schemas and Scripts

Within an emotional context that highlights deficiency, it is little wonder that schemas and scripts of the self are characterized by self-criticism (Shahar, 2015b). It is also not surprising that the other toward which the self is attuning is schematized as punitive and judgmental (Mongrain, 1998). The author has posited this previously (Shahar, 2018) but would now like to go beyond his previous contribution by here imbuing self and others with *agency*^3^. Namely, self and others are schematized and scripted in the psyche as acting upon each other (Shahar, 2004, 2010). The other conveys judgment and disappointment, while the self is actively attempting to shield against these punitive judgments and disappointments in two ways: appeasing the other and protesting against the other. Appeasement is done by way of what Klein calls “reparation” (see Thieberger, 1991): a fantasy of reconstituting the other as good is accompanied by solicitous behavior. The self is trying to “be good” so that the other deems the self as non-deficient. Protests come to the fore either when the self does not succeed in appeasing the other, or by way of a “preemptive strike”: “Are you mad at me?” The tone of the latter question is angry rather than inquisitive. It is better understood as “how dare you be mad at me when I am so good!”

In addition, there is a subtle experience of the other as seductive, in an evaluative, rather than erotic, sense. The seduction may be represented by the following unsaid words: “If you only accomplish this or that, be this or that, then I will cease judging you and will lovingly accept what you are.” Such seduction is, of course, intimately tied to the subtle hope described above, which dwells within the affective spectrum of the person.

### Time Axis

As the author has shown in prior sections, all three time points are included in the reformulated depressive position. The past produces storage of autobiographical memories from which the person draws the experience of being wronged (maltreated) by the other, but also the possibility of being, at least, partly responsible for the wrongdoing because the person somehow offended the other (Ferenczi, 1933). Hence: “You have hurt me. Had I hurt you before?”

In the present, there are active exchanges, both internally and externally, between self and others that revolve around hurt, grievance, and wrongdoing: Someone is always hurting someone else. These schemas and scripts surface but are also amalgamated by the aforementioned regulatory maneuvers, shifting blame from self to other and *vice versa*. Thus: “You are hurting me. Do I deserve it?”

Following Sullivan (1953) and Summers (2003), however, the author, here, highlights the role of the future, where both dread and hope lie (Mitchell, 1995b). The self yearns for an experience of an accepting other but dreads a scenario whereby, despite all efforts, the other will remain judgmental and punitive. The tragedy is that the self, *via* interpersonal action, actually solidifies this punitive judgment by evoking rejections, confrontations, and interpersonal loss (Buss, 1987).

### Interpersonal Action

This issue is, probably, the most important one in terms of bridging psychoanalytic theory and empirical psychology, as well as bridging clinical psychoanalysis and other therapeutic schools of thought. The tendency of depressed individuals to contribute to their own interpersonal strife is consensually agreed upon (Coyne, 1976a,b; Hammen, 1991; Joiner and Coyne, 1999; Joiner, 2000). One of the great strengths of psychoanalysis in general, and of object relations theory in particular, is their ability to give life to interpersonal descriptions of depression by portraying the inner drama that underlies social exchanges (Mitchell, 1995a). Horney (1937) called this *externalization*, positing that it serves as the basis of the *vicious cycle*. Both terms play a major role in writings of Wachtel about *cyclical psychoanalysis*. While most treatments of externalization and the vicious cycle focus on the present, the author proposes to extend these into the future. Depressed individuals project into their future both their hope for an accepting other as well as their expectations of a judgmental and punitive one. Their actions, however, automated throughout the years, tend to be more consistent with the latter than the former. In a manner consistent with the concept of Klein of projective identification but also with role responsiveness of Sandler (1976), their actions exert pressure on others to react negatively rather than with compassion: “Are you mad at me?”; “No, I am not”; “Yes, you are!”; “Well, now I am.”

## Contribution of RORT to Understanding and Treating Suicidal Depression

The issue of suicide is much broader than that of suicidal depression. As a global public health problem (World Health Organization, 2014), suicide stems from a wide array of psychopathologies. Nevertheless, mood disorders are prominent within this array (Henriksson et al., 1993; Bertolote et al., 2003; Dumais et al., 2005), and major depressive disorder (MDD), in particular, has been a particularly salient risk factor (Malone et al., 1995). However, there are many patients with MDD who do not eventually attempt suicide, rendering suicidal depression a riddle in urgent need of solving.

Let us now rely on the reformulated depressive position and see how it sheds light on suicidal depression. Depressed individuals project their self-with-other representations onto the future (see Figure 1B). They expect (dread) that others will hurt (criticize) them, but, at the same time, they hope that these others will actually heal them, that they will accept them as they are. However, as noted above, their interpersonal behavior is consistent with their dread, rather than their hope (Mitchell, 1995b): In an attempt to counteract deficiency, they project criticism onto others, thereby provoking rejection and loss. The latter activate self-criticism, demeaning affect, and the punitive internal other. This is employed alongside the aforementioned downregulation of authenticity, which, using terms of Winnicott, diminishes the true self and bolsters the *false self*. Hope is still maintained *via* compulsive purposefulness, but the latter depletes ego resources. Ultimately, hope is replaced with frustration and agitation, which further traps the person in interpersonal turmoil, perhaps to an irreversible point. The internal other thus becomes more persecutory, necessitating its physical annihilation. The author holds, in other words, that suicide is really murder-suicide.

These postulates have important implications for the understanding and treatment of suicidal depression. The author enumerates these implications below.

### Implications for the Understanding of Suicidal Depression

1. The deterioration from “uncomplicated depression” to suicidal depression is most noticeable in the interpersonal arena. However,
2. This arena interacts with a complex mental structure, such that the intrapersonal and interpersonal are reciprocally causative.
3. The deterioration is likely to be both exhaustive—encompassing virtually all key relationships—and relatively fast (i.e., involving the severing of one relationship after the other).
4. The crux of the exchange between person and context is the presence of gnawing deficiency and the inability to tolerate it [see Zetzel (1964) for a discussion of the inability to tolerate depression].

### Implications for the Treatment of Suicidal Depression

1. The transference-countertransference matrix is a crucial arena for identifying, understanding, and diffusing the deterioration to suicidal depression. Namely, patients will do everything within their power to evoke a sense of deficiency into the therapist and the therapeutic relationship.
2. At the same time, the patient's harbor hope that the therapist will not retaliate (Winnicott, 1969), whereby retaliation may be manifested by either crumbling or attacking back.
3. By not retaliating, therapists “hold” the future for their patients.
4. Holding the future (Shahar and Schiller, 2016b) inevitably involves the use of both psychoanalytic understanding and techniques (e.g., interpretation) as well as active techniques aimed at assisting the patients in refraining from begetting vicious interpersonal cycles.
5. Efforts geared toward helping the patients refrain from begetting vicious cycles should be made early in the treatment process. Otherwise, the link between the inner world (the depressive position) and the interpersonal arena might maladaptively amalgamate.
6. Use of active techniques can and should be employed to bolster psychodynamic exploration, and *vice versa*: Psychodynamic exploration can and should set the stage for the employment of active techniques (Shahar and Govrin, 2017).

Points 4 through 6 above are particularly pertinent to the aim of the present article, which seeks to build a dialog between clinical psychoanalysis and other therapeutic schools of thought. I will, therefore, elaborate on these points by illustrating the psychodynamic use of a very potent active technique for treating suicidal depression: behavioral activation (BA). Inspired by behaviorism and social-cognitive theories (Dimidjian et al., 2011), BA consists of systematically encouraging patients to pursue pleasurable and meaningful daily activities *in the face of* depressive anhedonia (i.e., the absence of positive affect). This strategy “reboots” the neurobiological reward system underlying operant conditioning, thereby alleviating anhedonia and, in turn, other depressive symptoms. Rigorous studies demonstrate that BA is superior to “standard” CBT in terms of improving depressive outcomes (Dimidjian et al., 2011).

Aner Govrin and I (Shahar and Govrin, 2017) have presented a conceptual framework for employing active techniques, primarily BA, in a way that not only reduces depressive symptoms but also bolsters psychoanalytic and existential growth and gain. Specifically, we argued that by systematically encouraging patients to schedule pleasurable/meaningful activities in their daily routine, BA not only “reboots” the reward system but also increases awareness of the patients of their various self-concept aspects and representations of other people, because the patients essentially expose themselves to a variety of people and social environments. Moreover, when the patients execute activities even though they “do not feel like acting,” they strengthen their ego resources and adaptive defense mechanisms. Because pleasurable and meaningful activities are often done with others, BA assists the patients in establishing their own “holding environment.” The goal-directed nature of these activities fosters hope. Pleasurable and meaningful activities also facilitate existential values such as responsibility, participation (being in the world), and transcendence over rumination and worries. In our work, Govrin and the author emphasized that these potential psychoanalytic and existential gains can only ensue if the therapist targets them explicitly and discusses them with the patient.

Could such a psychoanalytic and existential use of BA be also relevant to suicidal depression? The answer of the author is “yes,” and to demonstrate it, he refers to Figure 1B. Considering *affect* first, positive activities foster emotions, such as joy and enthusiasm, which are likely to dilute demeaning, criticism-based affect. Curiosity, another emotion likely to dilute demeaning affect, is defined as “a positive emotional-motivational system associated with the recognition, pursuit, and self-regulation of novel and challenging opportunities” (Kashdan and Fincham, 2004, p. 291), and considered a strong protective factor (Kashdan, 2004). In a fascinating study on military veterans with suicidal ideation, Denneson et al. (2017) found that curiosity reduced the strength of the associations between distress and suicide ideations. Turning to affect regulation, the very same study by Denneson et al. (2017) showed that curiosity *prospectively* weakened the effect of distress on increased coping efficacy to stop negative thoughts. Thus, positive affect, such as joy, enthusiasm, and curiosity, strengthens defenses and improves cognition and reality testing, thereby minimizing the adverse effect of counteracting deficiency. These emotions work against downregulating authenticity, and, instead of making purposefulness compulsive, they transform goal-directed action into *play* (Winnicott, 1989). Moving on to schemas and scripts, ability of patients to produce pleasurable/meaningful activities in the face of the tyranny of negative affect is inconsistent with a deficient self and is highly consistent with a realistically based positive sense of self, that these activities are done with others increases accessibility to “good objects” (i.e., positive schemas of other people) in a way that echoes what Blatt and Auerbach call “adaptive projective identification” (Blatt et al., 1996; Auerbach and Blatt, 2001). This is also likely to occur within the transference-countertransference matrix, as the joint therapeutic effort to identify pleasurable/meaningful activities and to support patients in scheduling and employing them may infuse the therapeutic alliance with a sense of comradery and mutual gratitude. All of these adaptive outcomes concerning affect, affect regulation, and schemas/scripts constitute a counter vector to the toxic one depicted in Figure 1B. This counter vector is manifested not only *via* inner events (thoughts, affects) but also *via* interpersonal action (establishing and maintaining nurturing relationships alongside creating interpersonal strife). The experience of the time axis, primarily the future, is also influenced: “It is not the other who is going to heal me. It is I who is going to do so, partly by creating a benevolent other.”

One caveat is in order here. Just as in the case of uncomplicated depression, all of the benefits of BA for the treatment of suicidal depression are contingent upon the willingness of the therapist (and skill) in engaging the patient in a discussion of these issues. Thus, we may say, in faithfulness to seminal legacy of Wachtel (1987, 2014), that insight can and should follow action but also *vice versa*.

## Conclusion

The author opened this article, lamenting the sordid state of affairs with respect to the seclusion of (clinical) psychoanalysis from prominent strands in psychology, psychiatry, psychopathology, and psychotherapy, and noted the hostility and ignorance of psychoanalysis toward the social-cognitive nomenclature. Next, he presented RORT and demonstrated how, using this nomenclature, work of Melanie Klein on the positions could be brought to life in a manner that is more scientifically accurate and appealing to people outside of psychoanalysis. He then applied RORT to the understanding of depression in general (the reformulated depressive position) and suicidal depression, in particular, showing how such reformulation is potentially useful, not only in terms of shedding light on suicidal depression but also in treating it psychoanalytically using techniques (e.g., BA), borrowed from other therapeutic schools of thoughts (e.g., CBT). When this is accomplished, psychoanalysis not only usefully assimilates other approaches into its midst but also accommodates them, in turn allowing for its own development and growth.^4^

Reflecting on my own approach to psychotherapy, RORT may be seen as a special form of theoretical integration, which the author titles *accomodative integration*. It is theoretical because it relies on ORT but also on existentialism and cognitive research. However, in contrast to assimilative integration, in which the external influences are peripheral to the original therapeutic persuasion, in RORT, the external influences (existentialism and cognitive psychology) repeatedly transform the original persuasion (ORT) and propel the latter to *accommodate* the former.

## Data Availability Statement

The original contributions presented in the study are included in the article/supplementary material, further inquiries can be directed to the corresponding author/s.

## Author Contributions

The author confirms being the sole contributor of this work and has approved it for publication.

## Conflict of Interest

The author declares that the research was conducted in the absence of any commercial or financial relationships that could be construed as a potential conflict of interest.

## Publisher's Note

All claims expressed in this article are solely those of the authors and do not necessarily represent those of their affiliated organizations, or those of the publisher, the editors and the reviewers. Any product that may be evaluated in this article, or claim that may be made by its manufacturer, is not guaranteed or endorsed by the publisher.

## Footnotes

^1^When manualized psychodynamic/psychoanalytic therapeutic packages are included within such guidelines, they resemble non-psychoanalytic therapeutic manuals in many respects (e.g., a clear structure, inclusion of psychoeducation, clear therapeutic goals, and assessment of symptoms). The extent to which such manualized psychodynamic/psychoanalytic therapeutic packages are on a continuum with psychoanalytic/psychodynamic treatment that is practiced outside scientific settings is unclear.

^2^Diluting the boundaries between conscious and unconscious materials in general, and defense mechanism in particular, brings to the fore notion of S. Freud (1895) of disavowal, which he used as an alternative to denial. There is a dispute within the psychoanalytic literature as to the exact meaning of disavowal and – relatively – whether it is a defense mechanism (Zepf, 2013). It seems, however, that there is a consensus that, in disavowal, the person dims awareness of an external fact so as to maintain inner coherence (Priel, 1991; Shahar and Schiller, 2016b). Whether it may be deemed a defense mechanism or not appears to be contingent upon the extent to which disavowal is conscious, because S. Freud, who was vague with respect to this issue, considered defense mechanisms as unconscious (but see Vaillant, 1992). From the position proposed here, it is of little importance of whether disavowal is conscious or not, a defense mechanism (in the Freudian sense) or not. It is deemed an important affect regulatory mechanism, and we consider it to be central to the psyche and extensively used.

^3^The author has previously proposed replacing “object relations” with “agents in relations (AIR)” in order to capture the agentic, goal-directed nature of interpersonal relations and mental representations of self with others (Shahar, 2010).

^4^The psychotherapy integration movement stipulates four forms of psychotherapy integration (Ziv-Beiman and Shahar, 2015). *Theoretical integration* consists of a clear, albeit integrated, theoretical position concerning human development, personality, psychopathology, and psychotherapy, from which techniques are derived. *Eclecticism* amounts to matching interventions with the problems of patients irrespective of theory. *Common-facto*rs *integration* refers to offering interventions that have been shown to be effective in all strands of therapy (e.g., empathy). Finally, *assimilative integration* pertains to adherence to a single theoretical approach, while assimilating interventions from other strands of psychotherapy into it. An example would be a self-proclaimed psychodynamic psychotherapist who will occasionally use CBT techniques.

## References

[B1] Ainsworth M. D. S. Bell S. M. (1970). Attachment, exploration, and separation: Illustrated by the behavior of one-year-olds in a strange situation. Child Dev. 41, 49–67. 10.2307/1127388 5490680

[B2] Amati D. Shallice T. (2007). On the emergence of modern humans. Cognition 103, 358–385. 10.1016/j.cognition.2006.04.002 16709406

[B3] Auerbach J. S. Blatt S. J. (2001). Self-reflexivity, intersubjectivity, and therapeutic change. Psychoanal. Psychol. 18, 427–450. 10.1037//0736-9735.18.3.427

[B4] Austin J. T. Vancouver J. B. (1996). Goal constructs in psychology: Structure, process, and content. Psychol. Bull. 120, 338–375. 10.1037/0033-2909.120.3.338

[B5] Bandura A. (1986). Social Foundations of Thought and Action: A Social Cognitive Theory. Prentice-Hall, Inc.

[B6] Barett T. F. (2006). Manic defenses against loneliness in adolescence. Psychoanal. Study Child 63, 111–136. 10.1080/00797308.2008.11800801 19449791

[B7] Bartholomew K. Horowitz L. M. (1991). Attachment styles among young adults: A test of a four category model. J. Personal. Soc. Psychol. 61, 226–244. 10.1037/0022-3514.61.2.226 1920064

[B8] Beck A. T. (1996). Beyond belief: A theory of modes, personality, and psychopathology, in Frontiers of Cognitive Therapy, ed Salkovskis P. M. (New York, NY: Guilford).

[B9] Bertolote J. M. Fleischmann A. De Leo D. Wasserman D. (2003). Suicide and mental disorders: Do we know enough? Br. J. Psychiatry 183, 382–383. 10.1192/bjp.183.5.382 14594911

[B10] Besser A. (2004). Self- and best friend assessments of personality vulnerability and defenses in the prediction of depression. Soc. Behav. Pers. 32, 559–594. 10.2224/sbp.2004.32.6.559

[B11] Beutel M. E. Greenberg L. Lane R. D. Subic-Wrana C. (2019). Treating anxiety disorders by emotion-focused psychodynamic psychotherapy (EFPP)-An integrative, transdiagnostic approach. Clin. Psychol. Psychother. 26, 1–13. 10.1002/cpp.2325 30255535

[B12] Billig M. (1999). Freudian Repression: Conversationcreating the Unconscious. Cambridge: CambridgeUniversity Press. 10.1017/CBO9780511490088

[B13] Blass R. B. (2010). Affirming 'That's not psycho-analysis!' On the value of the politically incorrect act of attempting to define the limits of our field. Int. J. Psychoanal. 91, 81–89. 10.1111/j.1745-8315.2009.00211.x 20433476

[B14] Blass R. B. Blatt S. J. (1992). Attachment and separateness-A theoretical context for the integration of object relations theory with self-psychology. Psychoanalytic Study Child 47, 189–203. 10.1080/00797308.1992.11822671 1289929

[B15] Blass R. B. Carmeli Z. (2007). The case against neuropsychoanalysis: On fallacies underlying psychoanalysis' latest scientific trend and its negative impact on psychoanalytic discourse. Int. J. Psychoanal. 88, 19–40. 10.1516/6NCA-A4MA-MFQ7-0JTJ 17244565

[B16] Blatt S. J. (1990). The Rorschach: A test of perception or an evaluation of representation. J. Pers. Assess. 55, 394–416. 10.1207/s15327752jpa5503&4_1 2280313

[B17] Blatt S. J. (1995a). Representational structures in psychopathology, in Rochester Symposium on Developmental Psychopathology, Vol. 6. Emotion, Cognition, and Representation, eds Cicchetti D. Toth S. L. (Rochester, NY: University of Rochester Press).

[B18] Blatt S. J. (1995b). The destructiveness of perfectionism: Implications for the treatment of depression. Am. Psychol. 50, 1003–1020. 10.1037/0003-066X.50.12.1003 8561378

[B19] Blatt S. J. Auerbach J. S. Levy K. N. (1997). Mental representations in personality development, psychopathology, and the therapeutic process. Rev. Gene. Psychol. 1, 351–374. 10.1037/1089-2680.1.4.35111379719

[B20] Blatt S. J. Levy K. N. (2003). Attachment theory, psychoanalysis, personality development, and psychopathology. Psychoanal. Inquiry 23, 102–150. 10.1080/07351692309349028

[B21] Blatt S. J. Stayner D. Auerbach J. S. Behrends R. S. (1996). Change in object and self-representations in long-term, intensive, inpatient treatment of seriously disturbed adolescents and young adults. Psychiatry 59, 82–107. 10.1080/00332747.1996.11024752 8744640

[B22] Blumental J. A. Lett H. S. (2003). Depression as a risk factor for mortality after coronary artery bypass surgery. Lancet. 362, 604–609. 10.1016/S0140-6736(03)14190-6 12944059

[B23] Bornstein R. F. (2001). The impending death of psychoanalysis. Psychoanal. Psychol. 18, 3–20. 10.1037/0736-9735.18.1.2

[B24] Bornstein R. F. (2007). Nomothetic psychoanalysis. Psychoanal. Psychol. 24, 590–602. 10.1037/0736-9735.24.4.590

[B25] Bowlby J. (1969). Attachment. Attachment and Loss: Vol. 1. Loss. New York, NY: Basic Books.

[B26] Brown G. W. Harris T. (1978). The Social Origins of Depression: A Study of Psychiatric Disorder in Women. New York, NY: Free Press.

[B27] Buss D. M. (1987). Selection, evocation, and manipulation. J. Personal. Soc. Psychol. 53, 1214–1221. 10.1037/0022-3514.53.6.1214 3320336

[B28] Bylsma L. M. Taylor-Clift A. Rottenberg J. (2011). Emotional reactivity to daily events in major and minor depression. J. Abnorm. Psychol. 120, 155–167. 10.1037/a0021662 21319928

[B29] Cervone D. (1991). The two disciplines of personality psychology. Psychol. Sci. 2, 371–377. 10.1111/j.1467-9280.1991.tb00169.x

[B30] Cooper D. E. (1999). Existentialism. Oxford: Basil Blackwell.

[B31] Coyne J. C. (1976a). Towards an interactional description of depression. Psychiatry 39, 28–40. 10.1080/00332747.1976.11023874 1257353

[B32] Coyne J. C. (1976b). Depression and the response of others. J. Abnorm. Psychol. 85, 186–193. 10.1037/0021-843X.85.2.186 1254779

[B33] Coyne J. C. (1986). Ambiguity and controversy: An introduction, in Essential Papers on Depression, ed Coyne J. C. (New York, NY: New York University Press).

[B34] Cramer P. (2006). Protecting the Self: Defense Mechanisms in Action. New York, NY: Guilford.

[B35] Dalal F. (2019). CBT: The Cognitive-Behavioral Tsunami: Managerialism, Politics, and the Corruption of Science. London: Routledge. 10.4324/9780429457814 PMC6532820 31147336

[B36] Deci E. L. Ryan R. M. (1985). Intrinsic Motivation and Self-Determination in Human Behavior. New York, NY: Plenum. 10.1007/978-1-4899-2271-7

[B37] Deci E. L. Ryan R. M. (2000). The “what” and “why” of goal pursuits: Human needs and the self-determination of behavior. Psychol. Inq. 11, 227–268. 10.1207/S15327965PLI1104_01

[B38] Denneson L. M. Smolenski D. J. Bush N. E. Dubscha S. K. (2017). Curiosity improves coping efficacy and reduces suicidal ideation severity among military veterans at risk for suicide. Psychiatry Res. 249, 125–131. 10.1016/j.psychres.2017.01.018 28092792

[B39] Dew M. A. Bromet E. J. (1991). Effects of depression on social support in a community sample of women, in The Social Context of Coping, ed Eckenrode J. (Boston, MA: Springer). 10.1007/978-1-4899-3740-7_9

[B40] Dimidjian S. Barrera M. Jr. Martell C. Muñoz R. F. Lewinsohn P. M. (2011). The origins and current status of behavioral activation treatments for depression. Annu. Rev. Clin. Psychol. 7, 1–38. 10.1146/annurev-clinpsy-032210-104535 21275642

[B41] Dowds B. (2018). Depression and the Erosion of the Self in Late Modernity: The Lessons of ICARUS. London and New York, NY: Routledge. 10.4324/9780429452741

[B42] Dumais A. Lesage A. D. Alda M. Rouleau G. Dumont M. Chawky N.. (2005). Risk factors for suicide completion in major depression: a case-control study of impulsive and aggressive behaviors in men. Am. J. Psychiatry 162, 2116–2124.? 10.1176/appi.ajp.162.11.2116 16263852

[B43] Dunkley D. M. Zuroff D. C. Blankstein K. R. (2003). Self-critical perfectionism and daily affect: Dispositional and situational influences on stress and coping. J. Personal. Soc. Psychol. 84, 234–252. 10.1037/0022-3514.84.1.234 12518982

[B44] Eagle M. N. (2011). From Classical to Contemporary Psychoanalysis: A Critique and Integration. London and New York, NY: Routledge. 10.4324/9780203868553

[B45] Erdelyi M. H. (2006). The unified theory of repression. Behav. Brain Sci. 29, 499–511. 10.1017/S0140525X06009113 17156548

[B46] Ettman C. K. Abdalla S. M. Galea S. (2020). Prevalence of depression symptoms in US adults before and during the COVID-19 pandemic. JAMA Netw. Open 3:e2019686. 10.1001/jamanetworkopen.2020.19686 32876685 PMC7489837

[B47] Ferenczi S. (1933). Confusion of Tongues Between Adults and the Child. London: Hogarth.

[B48] Fink B. (2009). A Clinical Introduction to Lacanian Psychoanalysis: Theory and Technique. Harvard, MA: Harvard University Press.

[B49] Fonagy P. Allison E. (2012). What is mentalization? The concept and its foundations in developmental research, in Minding the Child: Mentalization-Based Interventions With Children, Young People and Their Families, eds Midgley N. Vrouva I. (Routledge/Taylor & Francis Group).

[B50] Fonagy P. Luyten P. (2018). Attachment, mentalizing, and the self, in Handbook of Personality Disorders: Theory, Research, and Treatment, eds Livesley W. J. Larstone R. (The Guilford Press).

[B51] Fonagy P. Luyten P. Allison E. Campbell C. (2018). Reconciling psychoanalytic ideas with attachment theory, in Handbook of Attachment, eds Shaver P. Cassidy J. (New York, NY: Guilford Press). 10.4324/9780429472060

[B52] Fosco G. M. Van Ryzin M. J. Connell A. M. Stormshak E. A. (2016). Preventing adolescent depression with the family check-up: Examining family conflict as a mechanism of change. J. Family Psychol. 30, 82–92. 10.1037/fam0000147 26414418 PMC4742422

[B53] Freud S. (1895). Studies on hysteria. Stand. Edn. 2, 1–305.

[B54] Freud S. (1921). Group Psychology and the Analysis of the Ego. The Standard Edition of the Complete Psychological Works of Sigmund Freud, Volume XVIII (1920-1922): Beyond the Pleasure Principle. London: Group Psychology and Other Works.

[B55] Freud S. (1926). Inhibitions, symptoms and anxiety, in The Standard Edition of the Complete Psychological Works of Sigmund Freud, ed Strachey J. (London: Hogarth Press).

[B56] Freud S. (1930). Civilization and its Discontents. Hogarth.

[B57] Ghent E. (1989). Credo: The dialectics of one-person and two-person psychologies. Contemp. Psychoanal. 25, 169–211. 10.1080/00107530.1989.10746289

[B58] Green J. D. Sedikides C. Pinter B. Van Tongeren D. R. (2009). Two sides to self-protection: Self-improvement strivings and feedback from close relationships eliminate mnemic neglect. Self Identity 8, 233–250. 10.1080/15298860802505145

[B59] Greenberg J. R. Mitchell S. A. (1983). Object Relations in Psychoanalytic Theory. Cambridge: Harvard University Press. 10.2307/j.ctvjk2xv6

[B60] Guideline Development Panel for the Treatment of Depressive Disorders (2019). Clinical Practice Guidelines for the Treatment of Depression Across Three Age Cohorts. American Psychological Association.

[B61] Haan N. (1977). Coping and Defending. New York, NY: Academic.

[B62] Hammen C. (1991). The generation of stress in the course of unipolar depression. J. Abnorm. Psychol. 100, 555–561. 10.1037/0021-843X.100.4.555 1757669

[B63] Henriksson M. M. Aro H. M. Marttunen M. J. Heikkinen M. E. Isomets,ä E. T. Kuoppasalmi K. I.. (1993). Mental disorders and comorbidity in suicide. Am. J. Psychiatry 150, 935–940. 10.1176/ajp.150.6.935 8494072

[B64] Hoffman I. Z. (2009). Doublethinking our way to “scientific” legitimacy: The desiccation of human experience. J. Am. Psychoanal. Assoc. 57, 1043–1069. 10.1177/0003065109343925 19837853

[B65] Horney K. (1937). The Neurotic Personality of Our Time. New York, NY: Norton.

[B66] Horney K. (1950). Neurosis and Human Growth; The Struggle Toward Self Realization. New York, NY: W. W. Norton.

[B67] Joiner T. E. (1994). Contagious depression: Existence, specificity to depressive symptoms, and the role of reassurance seeking. J. Personal. Soc. Psychol. 67, 287–296. 10.1037/0022-3514.67.2.287 7932064

[B68] Joiner T. E. (2000). Depression's vicious scree: Self-propagating and erosive processes in depression chronicity. Clin. Psychol. Sci. Practice 7, 203–218. 10.1093/clipsy.7.2.203

[B69] Joiner T. E. (2007). Why People Die by Suicide. Cambridge, MA: Harvard University Press. 10.2307/j.ctvjghv2f

[B70] Joiner T. E. Coyne J. C. (1999). The Interactional Nature of Depression: Advances in Interpersonal Approaches. Washington, DC: American Psychological Association. 10.1037/10311-000

[B71] Joiner T. E. Metalsky J. I. Katz J. Beach S. R. H. (1999). Depression and excessive reassurance seeking. Psychol. Inq. 10, 269–278. 10.1207/S15327965PLI1004_1

[B72] Jurist E. Orfanos S. D. (2016). It takes two to tango: Introduction to the special issue on psychoanalysis and the humanities. Psychoanalytic Psychol. 33(Suppl 1), S1–S7. 10.1037/pap0000076

[B73] Kahneman D. (2011). Thinking, Fast and Slow. Farrar, Straus and Giroux.

[B74] Kashdan T. B. (2004). Curiosity, in Character Strengths and Virtues: A Handbook and Classification, eds Peterson C. Seligman M. E. P. (Washington DC: American Psychological Association and Oxford University Press).

[B75] Kashdan T. B. Fincham F. D. (2004). Facilitating curiosity: A social and self-regulatory perspective for scientifically based interventions, in: Positive Psychology in Practice, eds Linely P. A. Joseph S. (Hoboken NJ: John Wiley & Sons).

[B76] Kernberg O. F. (2006). The pressing need to increase research in and on psychoanalysis. Int. J. Psychoanaly. 87, 919–926. 10.1516/46N7-ULAM-DQKR-VGRT 16877244

[B77] Kessler R. C. Berglund P. Demler O. Jin R. Walters E. E. (2005). Lifetime prevalence and age-of-onset distributions of DSM-IV disorders in the National Comorbidity Survey Replication. Arch. Gen. Psychiatry 62, 593–602. 10.1001/archpsyc.62.6.593 15939837

[B78] Klein M. (1928). Early stages of the oedipus con?ict. Int. J. Psychoanaly. 9, 167–180.

[B79] Klein M. (1935). A contribution to the psychogenesis of manic-depressive states. Int. J. Psychoanaly. 15, 145–174. 20243429

[B80] Klein M. (1940). Mourning and its relation to manic-depressive states. Int. J. Psychoanaly. 21, 125–153.

[B81] Klein M. (1945). The Oedipus complex in the light of early anxieties. Int. J. Psychoanaly. 26, 11–33. 10.4324/9780429482601-2 21006521

[B82] Kohut H. (1980). Self-Psychology and the Humanities: Reflections on a New Psychoanalytic Approach. New York NY: Norton.

[B83] Kyle A. (2014). Intellectualization and its lookalikes. Psychoanal. Rev. 10, 615–632. 10.1521/prev.2014.101.5.615 25247283

[B84] Lépine J. P. Briley M. (2011). The increasing burden of depression. Neuropsychiatricdis. Treatment 7, 3–7. 10.2147/NDT.S19617 21750622 PMC3131101

[B85] Levy K. N. Blatt S. J. (1999). Attachment theory and psychoanalysis: Further differentiation within insecure attachment patterns. Psychoanaly. Inquiry 19, 541–575. 10.1080/07351699909534266

[B86] Levy K. N. Blatt S. J. Shaver P. (1998). Attachment styles and parental representations. J. Personal. Soc. Psychol. 74, 407–419. 10.1037/0022-3514.74.2.407

[B87] Levy K. N. Meehan K. B. Kelly K. M. Reynoso J. S. Clarkin J. F. Kernberg O. F. (2006). Change in attachment patterns and reflective function in a randomized control trial of transference-focused psychotherapy for borderline personality disorder. J. Consult. Clin. Psychol. 74, 1027–1040. 10.1037/0022-006X.74.6.1027 17154733

[B88] Main M. Kaplan N. Cassidy J. (1985). Security in infancy, childhood, and adulthood: A move to the level of representation. Monogr. Soc. Res. Child Dev. 50, 66–104. 10.2307/3333827

[B89] Malone K. M. Haas G. L. Sweeney J. A. Mann J. J. (1995). Major depression and the risk of attempted suicide. J. Affect. Disord. 34, 173–185. 10.1016/0165-0327(95)00015-F 7560545

[B90] May R. (1958). Contributions of existential psychotherapy, in Existence: A New Dimension in Psychiatry and Psychology, eds May R. Angel E. Ellenberger E. (New York, NY: Basic Books). 10.1037/11321-002

[B91] McDaniel M. A. Einstein G. O. (2007). Cognitive Psychology Program. Prospective Memory: An Overview and Synthesis of an Emerging Field. Los Angeles, CA: Sage Publications, Inc.

[B92] Mikulincer M. Shaver P. (2016). Attachment in Adulthood: Structure, Dynamics,and Change. New York, NY: Guilford.

[B93] Miller G. A. (1951). Language and Communication. New York NY: McGraw-Hill. 10.1037/11135-000

[B94] Miller G. A. (2003). The cognitive revolution: A historical perspective. Trends Cognit. Sci. 7, 141–144. 10.1016/S1364-6613(03)00029-9 12639696

[B95] Mischel W. (1973). Toward a cognitive social learning reconceptualization of personality. Psychol. Rev. 80, 252–283. 10.1037/h0035002 4721473

[B96] Mitchell S. A. (1995a). Interaction in the Kleinian and interpersonal traditions. Contemp. Psychoanal. 59, 65–91. 10.1080/00107530.1995.10746896

[B97] Mitchell S. A. (1995b). Hope and Dread in Psychoanalysis. New York, NY: Basic Books.

[B98] Mongrain M. (1998). Parental representations and support-seeking behaviors related to dependency and self-criticism. J. Pers. 66, 151–173. 10.1111/1467-6494.00007 9529661

[B99] Monroe S. M. Anderson S. F. (2015). Depression: The shroud of heterogeneity. Curr. Direct. Psychol. Sci. 24, 227–231. 10.1177/0963721414568342

[B100] Moroz M. Dunkley D. (2019). Self-critical perfectionism, experiential avoidance, and depressive and anxious symptoms over two-years: A three-wave longitudinal study. Behav. Res. Therapy 112, 18–27. 10.1016/j.brat.2018.11.006 30466031

[B101] Ogden T. H. (1992). The Matrix of the Mind: Object Relations and the Psychoanalytic Dialogue. London: Karnac.

[B102] Paul R. A. (1989). Psychoanalytic anthropology. Annu. Rev. Anthropol. 18, 177–202. 10.1146/annurev.an.18.100189.001141

[B103] Paykel E. S. (2003). Life events and affective disorders. Acta Psychiatr. Scand. 418, 61–66. 10.1034/j.1600-0447.108.s418.13.x 12956817

[B104] Pettit J. W. Joiner T. E. (2006). Chronic Depression: Interpersonal Sources, Therapeutic Solutions. Washington, DC: American Psychological Association Press. 10.1037/11291-000

[B105] Powell S. (2010). Psychoanalysis and the study of political science, in Grand Theories and Ideologies in the Social Sciences, ed Wiarda H. J. (New York, NY: Palgrave Macmillan).

[B106] Priel B. (1991). Disavowal in fiction. Int. Rev. Psycho-Analysis 18, 19–26.

[B107] Refaeli E. Bornstein D. Young J. (2010). Schema Therapy: Distinctive Features. London: Routledge. 10.4324/9780203841709

[B108] Ricœur P. (1970). Freud and Philosophy: An Essay on Interpretation. New Haven, CT: Yale University Press.

[B109] Rogers C. R. (1963). Actualizing tendency in relation to Motives and to consciousness, in Nebraska Symposium on Motivation, ed Jones M. R. (Lincolin, NE: U. Nebraska Press).

[B110] Rottenberg J. (2014). The Depths: The Evolutionary Basis of the Depression Epidemic. New York, NY: Basic Books.

[B111] Ryan R. M. Deci E. L. (2000). Self-determination theory and the facilitation of intrinsic motivation, social development, and well-being. Am. Psychol. 55, 68–78. 10.1037/0003-066X.55.1.68 11392867

[B112] Sandler J. (1976). Countertransference and role responsiveness. Int. Rev. Psychoanal. 3, 43–47.

[B113] Schafer R. (1954). Psychoanalytic Interpretation in Rorschach Testing. New York, NY: Grune& Stratton.

[B114] Schiller M. Hammen C. C. Shahar G. (2016). Comparing three theoretical models of the links between self, stress, and psychopathological Distress during emerging adulthood. Self Identity 15, 302–326. 10.1080/15298868.2015.1131736

[B115] Sedikides C. Green J. D. (2004). What I don't recall can't hurt me: Negativity versus information inconsistency as determinants of memorial self-defense. Soc. Cogn. 22, 4–29. 10.1521/soco.22.1.4.30987

[B116] Sedikides C. Green J. D. (2009). Memory as a self-protective mechanism. Soc. Personal. Psychol. Compass 3, 1055–1068. 10.1111/j.1751-9004.2009.00220.x

[B117] Seligman M. E. Railton P. Baumeister R. F. Sripada C. (2013). Navigating into the future or driven by the past. Perspect. Psychol. Sci. 8, 119–141. 10.1177/1745691612474317 26172493

[B118] Shahar G. (2001). Personality, shame, and the breakdown of social ties: The voice of quantitative depression research. Psychiatry 64, 229–238. 10.1521/psyc.64.3.228.18463 11708047

[B119] Shahar G. (2004). Transference-countertransference: Where the (political) action is. J. Psychother. Integr. 14, 371–396. 10.1037/1053-0479.14.4.371

[B120] Shahar G. (2006a). Clinical action: Introduction to the special section on the action perspective in clinical psychology. J. Clin. Psychol. 29, 1053–1064. 10.1002/jclp.20290 16810665

[B121] Shahar G. (2006b). Repression, suppression, and oppression (in depression). Behav. Brain Sci. 29:533. 10.1017/S0140525X06459112

[B122] Shahar G. (2010). Poetics, pragmatics, schematics, and the psychoanalysis-research dialogue (rift). Psychoanal. Psychother. 24, 315–328. 10.1080/02668734.2010.513544

[B123] Shahar G. (2011). Projectuality vs. eventuality: Sullivan, the ambivalent intentionalist. J. Psychother. Integ. 21, 211–220. 10.1037/a0022909

[B124] Shahar G. (2012). “I don't want to be here”: Projectuality and eventuality in Ms. T's case. J. Psychother. Integrat. 22, 27–32. 10.1037/a0027321

[B125] Shahar G. (2015a). Object relations theory. In The Encyclopedia of Clinical Psychology, eds Cautin R. Lillinfeld S. (New York, NY: Wiley). 10.1002/9781118625392.wbecp297

[B126] Shahar G. (2015b). Erosion: The Psychopathology of Self-Criticism. New York, NY: Oxford University Press. 10.1093/med:psych/9780199929368.001.0001

[B127] Shahar G. (2016). Criticism in the self, brain, social relations and social structure: Implications to psychodynamic psychiatry. Psychodyn. Psychiatry 44, 395–421. 10.1521/pdps.2016.44.3.395 27603804

[B128] Shahar G. (2018). The (suicidal-) depressive position. A novel, scientifically-informed reformulation. Psychodynamic Psychiatry 46, 265–293. 10.1521/pdps.2018.46.2.265 29809115

[B129] Shahar G. Bauminger R. Itamar S. (2020). A lion's blues: Heroism, heroic self-representations, and emotional distress in the life and character of Yonatan (Yoni) Netanyahu, Heroism. Science 5:5. 10.26736/hs.2020.02.05

[B130] Shahar G. Blatt S. J. Zuroff D. C. Krupnick J. Sotsky S. M. (2004a). Perfectionism impedes social relations and response to brief treatment for depression. J. Soc. Clin. Psychol. 23, 140–154. 10.1521/jscp.23.2.140.31017

[B131] Shahar G. Cross L. W. Henrich C. C. (2004b). Representations in action (Or: action models of development meet psychoanalytic conceptualization of mental representations). Psychoanal. Study Child 59, 261–293. 10.1080/00797308.2004.11800741 16240615

[B132] Shahar G. Davidson L. (2009). Participation-engagement: A philosophically-based heuristic for prioritizing interventions in the treatment of comorbid, complex, and chronic psychiatric conditions. Psychiatry 72, 154–176. 10.1521/psyc.2009.72.2.154 19614554

[B133] Shahar G. Govrin A. (2017). Psychodynamizing and existentializing cognitive-behavioral interventions: The case of Behavioral Activation (BA). Psychotherapy 54, 267–272. 10.1037/pst0000115 28758763

[B134] Shahar G. Henrich C. C. (2019). Role of adolescent exposure to missile attacks in the links between personality and psychopathology. Dev. Psychopathol. 31, 1367–1380. 10.1017/S0954579418000792 30520399

[B135] Shahar G. Henrich C. C. Blatt S. J. Ryan R. Little T. D. (2003). Interpersonal relatedness, self-definition, and their motivational orientation during adolescence: A theoretical and empirical integration. Dev. Psychol. 39, 470–483. 10.1037/0012-1649.39.3.470 12760516

[B136] Shahar G. Priel B. (2003). Active vulnerability, adolescent distress, and the mediating/suppressing role of life events. Personal. Individ. Differ. 35, 199–218. 10.1016/S0191-8869(02)00185-X

[B137] Shahar G. Schiller M. (2016a). A conqueror by stealth: Introduction to the special issue on humanism, existentialism, and psychotherapy integration. J. Psychother. Integr. 26, 1–4. 10.1037/int0000024

[B138] Shahar G. Schiller M. (2016b). Treating the depressive self: A psychodynamic-integrative approach, in The Self in Understanding and Treating Psychological Disorders, eds Kyrios K. Bhar S. Doron G. Mikulincer M. Roulding R. Nedeljkovic M. (Cambridge: Cambridge University Press).

[B139] Shulman S. Kalnitzki E. Shahar G. (2009). Meeting developmental challenges during emerging adulthood: The role of personality and social resources. J. Adolesc. Res. 24, 242–267. 10.1177/0743558408329303

[B140] Skinner B. F. (1957). Verbal Behavior. New York, NY: Appleton-Century-Crofts. 10.1037/11256-000

[B141] Skinner B. F. (1971). Beyond Freedom and Dignity. New York, NY: Knopf/Random House.

[B142] Stolorow R. D. Brandshaft B. Atwood G. E. (1987). Psychoanalytic Treatment: An Intersubjective Approach. Hillsdale, NJ: Analytic.

[B143] Strenger C. (1989). The classic and romantic vision in psychoanalysis. Int. J. Psychoanaly. 70, 593–610. 2606597

[B144] Sullivan H. S. (1953). The Interpersonal Theory of Psychiatry. W W Norton & Co.

[B145] Summers F. (2003). The future as intrinsic to the psyche and psychoanalytic therapy. Contemp. Psychoanal. 39:135153. 10.1080/00107530.2003.10747206

[B146] Swedish National Audit Office (2015). Summary: The Rehabilitation Guarantee is Not Working - Rethink or Discontinue. Available online at: https://www.riksrevisionen.se/download/18.78ae827d1605526e94b32d99/1518435460032/Summary_2015_19.pdf

[B147] Thieberger J. (1991). The concept of reparation in Melanie Klein's writing. Melanie Klein Object Relat. 9, 32–46.

[B148] Tuccitto D. E. Giacobbi P. R. Leite W. L. (2010). The internal structure of positive and negative affect: a confirmatory factor analysis of the PANAS. Educ. Psychol. Measure. 70, 125–141. 10.1177/0013164409344522

[B149] Tzelgov J. (1997). Automatic but conscious: That is how I act most of the time, in The Automaticity of Everyday Life, ed Wyer R. S. (Mahwah, NJ: Erlbaum).

[B150] Vaillant G. E. (1992). The historical origins and future potentialof Sigmund Freud's concept of the mechanisms of defense. Int. Rev. Psychoanal. 19, 36–50.

[B151] Vaillant G. E. (2011). Involuntary coping mechanisms: A psychodynamic perspectives. Dial. Clin. Neurosci. 13, 366–370. 10.31887/DCNS.2011.13.2/gvaillant PMC3182012 22034454

[B152] Wachtel P. L. (1987). Action and Insight. New York, NY: Guilford Press.

[B153] Wachtel P. L. (1994). Cyclical processes in personality and psychopathology. J. Abnorm. Psychol. 103, 51–54. 10.1037/0021-843X.103.1.51 8040480

[B154] Wachtel P. L. (2014). Cyclical Psychodynamics and the Contextual Self: The Inner World, the Intimate World, and the World of Culture and Society. London: Routledge. 10.4324/9781315794037

[B155] Watson D. Clark L. A. Tellegen A. (1988). Development and validation of brief measures of positive and negative affect: The PANAS scales. J. Personal. Soc. Psychol. 47, 1063–1070. 10.1037/0022-3514.54.6.1063 3397865

[B156] Westen D. (1991). Social cognition and object relations. Psychol. Bull. 109, 429–455. 10.1037/0033-2909.109.3.429

[B157] Westen D. (1998). The scientific legacy of Sigmund Freud: Toward a psychodynamically informed psychological science. Psychol. Bull. 124, 333–371. 10.1037/0033-2909.124.3.333

[B158] Whelton W. J. Greenberg L. S. (2005). Emotion in self-criticism. Pers. Individ. Dif. 38, 1583–1595. 10.1016/j.paid.2004.09.024

[B159] Winnicott D. W. (1958). Through Pediatrics to Psychoanalysis: Collected Papers, 1958. New York, NY: Basic Books.

[B160] Winnicott D. W. (1960). The theory of parent-infant relationships. Int. J. Psychoanaly. 41, 585–595. 13785877

[B161] Winnicott D. W. (1965). Ego Distortions in Terms of True and False Self. In the Maturational Process and the Facilitating Environment. London: Hogarth Press. 10.4324/9780429482410-12

[B162] Winnicott D. W. (1969). The use of the object. Int. J. Psychoanaly. 50, 711–716.

[B163] Winnicott D. W. (1989). Playing and Reality. London: Routledge.

[B164] World Health Organization (2012). Depression: A Global Crisis: World Mental Health Day. Available online at: http://www.who.int/mental_health/management/depression/wfmh_paper_depression_wmhd_2012.pdf (accessed October 10, 2012).

[B165] World Health Organization (2014). Preventing Suicide: A global imperative. Geneva: World Health Organization.

[B166] Yeomans F. E. Levy K. N. Caligor E. (2013). Transference-focused psychotherapy. Psychotherapy 50, 449–453. 10.1037/a0033417 24000869

[B167] Zepf S. (2013). A note on the application of the term “disavowal” in psychoanalysis. Scand Psychoanaly Rev. 36, 35–42. 10.1080/01062301.2013.795367

[B168] Zetzel E. (1964). Depression and the inability to bear it, in Drives, Affect and Behavior, ed Schur M. (International Universities Press).

[B169] Ziv-Beiman S. Shahar G. (2015). Psychotherapy integration, in The Encyclopedia of Clinical Psychology, eds Cautin R. Lilienfeld S. (New York, NY: Wiley).

